# Encapsulation of bougainvillea (*Bougainvillea spectabilis*) flower extract in *Urtica dioica* L. seed gum: Characterization, antioxidant/antimicrobial properties, and in vitro digestion

**DOI:** 10.1002/fsn3.2944

**Published:** 2022-06-23

**Authors:** Reza Esmaeilzadeh Kenari, Razie Razavi

**Affiliations:** ^1^ Department of Food Science and Technology Sari Agricultural Sciences and Natural Resources University Sari Iran

**Keywords:** bioactive compounds, bioavailability, controlled release, nanoemulsion, nanoparticle

## Abstract

*Bougainvillea spectabilis* extract (BSE), a rich source of bioactive compounds like phenolic, flavonoid, and anthocyanin, was used for encapsulation with *Urtica dioica* L. seed gum. The extract was obtained using shaking, bath, and probe ultrasound. The results showed that probe ultrasound extract was more efficient, as reflected by the higher value of total phenolic (4354.15 mg GAE/100 g FW), flavonoid (2431.25 mg CE/100 g FW), and anthocyanin content (106.57 mg CGE/100 g FW). BSE was encapsulated in *U. dioica* L. seed gum at 1:1 and 1:2 core to coating ratio. In both DPPH radical scavenging and FRAP assay, higher antioxidant activity was observed in the encapsulated extract than in the free extract. Encapsulated extracts exhibited 87.9 nm average diameter (polydispersity index below 0.23) and negative zeta potential. The average minimum inhibitory concentration (MIC) of nanoparticles against *Staphylococcus aureus*, *Escherichia coli*, *Listeria monocytogenes*, and *Salmonella enterica* was 112.5, 87.5, 112.5, and 87.5 μg/ml, respectively, while MIC of the free extract against *S. aureus* and *E. coli* was 150 and 125 μg/ml, respectively. The phenolic compounds are gradually released from the nanoparticles in the gastric and small intestine phase, whereas free extract released phenolic compounds quickly after entering the gastric phase. Considering antioxidant/antimicrobial activity and release properties, nanoparticles with a 1:1 ratio of core to coating had the potential to use as an effective natural preservative agent in food products besides the delivery of bioactive compounds to the human body.

## Graphical abstract

In this study for the first time, the extract of paper flower was extracted by ultrasound‐assisted extraction (bath and probe) and shaking method and extract nanoencapsulated in nettle seed gum as a new source of native and natural biopolymer. Our results showed encapsulation caused to enhance bioavailability of bioactive compounds of extract like phenolics, flavonoids, and tannin in the simulated gastrointestinal tract.

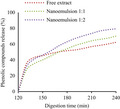

## 1 INTRODUCTION

The recent decades have witnessed a growing interest in reducing synthetic preservatives by replacing them with natural agents because of the public concern regarding the potential adverse effects like cancer, cardiovascular diseases, and diabetes (Bao et al., 2019; Orozco‐Villafuerte et al., 2018). The growing demand for natural antioxidant/antimicrobial compounds has triggered the extract characterization from a rich diversity source. *Bougainvillea spectabilis*, termed as “paper flower,” belongs to the family Nyctaginaceae, which is known as a vital medicine (Abarca‐Vargas et al., 2016; Alsamadany, 2020).

*Bougainvillea* species have been shown to possess phenolics, flavonoids, alkaloids, anthocyanin, tannins, saponins, phytate, and oxalate (Ikpeme et al., 2015; Singh, 2019). *Bougainvillea spectabilis* is a rich source of extraordinary secondary metabolites with several pharmacological properties like antibacterial, antioxidant, anticancer, antidiabetic, anti‐inflammatory, and antihyperlipidemic (Alsamadany, 2020; Compaore et al., 2013).

The extraction process is substantially the most vital step in obtaining valuable phytochemical compounds. The method of extraction interferes with the process of obtaining bioactive compounds (Abarca‐Vargas et al., 2016; Debiasi et al., 2021). Nowadays, the conventional methods of extraction have been replaced by green procedures like ultrasound‐assisted extraction due to its long extraction time, low extraction yield, degradation of bioactive compounds, and high solvent consumption (Debiasi et al., 2021; Razavi & Kenari, 2021).

Despite all the health benefits, anthocyanin and phenolics have been shown to have limited bioavailability after intake of a meal rich in them (Bao et al., 2019; Rahnemoon et al., 2021; Zhao et al., 2017). Also, they readily undergo rapid degradation under processing, storage, or gastrointestinal conditions due to pH, light, temperature, oxygen, and enzyme exposure (Bao et al., 2019; Huang et al., 2019; Razavi & Kenari, 2021; Zhao et al., 2017).

Encapsulating of bioactive compounds in diverse wall materials is a promising technology that can protect them in such conditions and improve their activity and bioavailability (Rahnemoon et al., 2021). Natural proteins, polysaccharides, and lipids are promising coating materials for the encapsulation of bioactive compounds which affects the stability and characteristics of final particles (Razavi & Kenari, 2021). Hydrocolloids are high‐molecular‐weight biopolymers with an extensive range of functions. Seeds are generally traditional source of different gums (Zamani & Razavi, 2021). Nettle (*Urtica dioica* L.) is a herb belonging to the Urticaceae family (Kutlu et al., 2020), which produces the most immense amount of mucilage (Zamani & Razavi, 2021). The rheological, functional, and physicochemical properties of nettle seed gum were studied before (Kutlu et al., 2020; Zamani & Razavi, 2021), but its usage possibilities in encapsulation have not been investigated before.

*Bougainvillea spectabilis* extract (BSE) can be encapsulated in colloidal dispersions with droplet sizes between 20 and 200 nm, referred to as a nanoemulsion. These systems efficiently support the use of bioactive compounds in food products by reducing the impact on taste, as well as by enhancing their antimicrobial/antioxidant activities and bioavailability (Seibert et al., 2019). Although the antimicrobial and antioxidant properties of BSE have been studied previously, the bioavailability and preservation potential of encapsulated BSE has not been previously investigated. Therefore, the objective of this study was to evaluate: (1) the effect of extraction method on bioactive compounds of BSE, (2) the antimicrobial and antioxidant activities of free and nanoencapsulated BSE, and (3) the efficiency of *U. dioica* L. seed gum in increasing the bioavailability of bioactive compounds in the simulated gastrointestinal tract.

## 2 MATERIALS AND METHODS

### 2.1 Materials

Air parts (flower) of *B. spectabilis* were collected from Sari in May 2021. Nettle seed was purchased from a local grocery. All reagents and chemicals were purchased from Sigma‐Aldrich.

### 2.2 Methods

#### 2.2.1 Extract production and extraction yield

The extracts of *B. spectabilis* flower were obtained using three different methods following the guidelines reported by Han et al. (2018) with slight modification. Five grams of the fine powder was added to 100 ml ethanol:water (50:50 V/V). The mixture was shaken in a shaker incubator (Unimax 1010, Heidolph) at 35°C for 1 h. For bath ultrasound extraction, the mixture was placed in an ultrasound water bath (Elma) at 35°C for 1 h (37 kHz–250 W). For probe ultrasound (PRO‐250, mLabs) extraction, the mixture was sonicated with a probe ultrasound set at 20 kHz (280 W) and 50% intensity at 35°C for 1 h. Then, all treatments were immediately centrifuged at 3000 rpm for 15 min, and supernatants were oven dried overnight at 45°C (Han et al., 2018). The extraction yield of extracts was calculated using the following equation:

(1) $$\text{Extraction yield}\;\left(\%\right)=\left(W_{1}/W_{2}\right)\times 100$$

W_1_ = the total weight of dried extracts obtained after drying and W_2_ = the total weight of ground flower taken for each extraction method (Abarca‐Vargas et al., 2016).

#### 2.2.2 Determination of the content of bioactive compounds and antioxidant activity

Total phenolic, flavonoid, and anthocyanin content of BSEs were determined using the method described by Kuspradini et al. (2016). Gallic acid, catechin, and cyanidin‐3‐glucoside were used as a standard to make a calibration curve, respectively (Kuspradini et al., 2016). Antioxidant activity of the extract was measured according to the DPPH radical scavenging activity and ferric reducing antioxidant activity (Razavi & Kenari, 2021).

#### 2.2.3 MIC and MBC determination

The assays were performed using two gram‐negative bacteria strains (*Escherichia coli* ATCC 10799 and *Salmonella enterica* ATCC 14028) and two gram‐positive bacteria (*Staphylococcus aureus* ATCC 33591 and *Listeria monocytogenes* ATCC 19115) cultivated in Mueller–Hinton broth (18 h at 37°C). For minimum inhibitory concentration (MIC) and minimum bactericidal concentration (MBC) evaluation, 0.5 McFarland (10^8^ CFU/ml) of different bacterial suspensions were prepared in sterile tubes containing peptone water and mixed with primary stock solution medium of Mueller–Hinton broth at 20 mg/L of concentration. Different dilutions of BSE in both free and nanoencapsulated forms were obtained by serial dilution and added to tubes at the same concentration. All tubes were incubated for 24 h at 37°C, and after that, each sample was cultured on Mueller–Hinton agar again and incubated at 37°C for 24 h. Finally, the growth or lack of development of bacteria in different concentrations for free and nanoencapsulated extract of *B. spectabilis* was observed, and MIC and MBC were determined.

#### 2.2.4 Extraction of nettle seed gum

Seed gum was provided under the condition described by Kutlu et al. (2020) with slight modification. Briefly, 100 g seeds were soaked in 1 L distilled water at 60°C for 1 h under a magnetic stirrer. Then, the mucilage separated from swollen seeds, passed through a vacuum filter, and oven dried at 45°C. The dried gum was milled and stored in a dry and cool place until use (Kutlu et al., 2020).

#### 2.2.5 Nanoemulsion preparation

Nanoemulsions were prepared by the method of Kenari et al. (2020) with slight modification. First, seed gum was dispersed in deionized water to achieve total solid content of 30%. The extract (25 mg) at 1000 ppm of concentration was added to 20 ml sunflower oil and 5 ml emulsifier Tween 80 and stirred for 2 h at 25°C until primary emulsion formed. Then, gum solution at two different ratios (1:1 and 1:2) of emulsion to gum solution was added to the emulsion gradually and homogenized using an ultraturrax (T25D, IKA) at 15,000 rpm and 10°C for 10 min (Kenari et al., 2020).

#### 2.2.6 Characterization of nanoemulsion

Particle size, polydispersity index (PDI), and zeta potential of nanoemulsions were evaluated by dynamic light scattering technology using Zetasizer (S90) instrument at 25°C. The nanoemulsion was diluted 20‐fold in deionized water to escape multiple scattering effects (Razavi et al., 2020). The encapsulation efficiency (EE) of BSE was calculated by measuring the amount of total phenolic compounds (TPC) of the extract before and after encapsulation. The ratio of unencapsulated TPC on TPC before encapsulation has been considered as EE (Rahnemoon et al., 2021). Nanoemulsions were dried using a freeze dryer (VaCO5, Zirbus) at 0.017 mPa and −57°C for 48 h. Then, the morphology of nanoemulsions was examined using a scanning electron microscope (Mira 3 LMU, Tescan, Czech Republic). For this purpose, samples were attached to aluminum stubs and covered with a thin layer of gold. The voltage device was 15 kV, and images were taken at 1000× magnification (Razavi et al., 2020).

#### 2.2.7 Simulated gastrointestinal condition

Simulated fluid for gastric (SGF) and small intestine (SIF) were prepared as described by Razavi et al. (2020). For the stomach digestion, 1.4 ml of SFG was mixed with 100 ml of nanoemulsion and put in a shaker incubator at 37°C for 2 h with continuous agitating at 100 rpm. For the intestinal digestion, 2.4 ml of SIF was mixed with 100 ml of digesta from the stomach stage and shook in a shaker incubator at 37°C for 2 h. In all experiments, the digesta was collected at 0, 30, 60, 90, and 120 min of digestion. After digestion, the samples were immediately centrifuged at 2000 rpm for 15 min, and after filtration, the filtrate was used to determine the content of bioactive compounds released from the nanoemulsion (Razavi et al., 2020).

### 2.3 Statistical analysis

The results were statistically analyzed by one‐way analysis of variance (ANOVA) with Duncan test (*p* < .05) and *T* test using SPSS version 20.0 statistical software (SPSS, Inc.). All experiments were carried out in triplicate.

## 3 RESULTS AND DISCUSSION

### 3.1 Extraction yield and content of bioactive compounds

Extraction is the first step in analyzing bioactive compounds from plant materials (Abarca‐Vargas et al., 2016). The results of extraction yield (EY) obtained by different methods are illustrated in Table 1. The EY ranged from 6.12% to 16.37%. The extract obtained by probe ultrasound showed higher EY followed by bath ultrasound and shaking. Kuspradini et al. (2016) reported extract yields between 5.80% and 13.30% for different flower extracts, which was lower than the amount obtained in this study. Debiasi et al. (2021) compared the EY of *B. spectabilis* white bracts with conventional technique of the extraction by maceration and ultrasound‐assisted technique at different extraction times. They revealed that the highest yield was presented by ultrasound (4.98%), which is in line with the results of this study. Abarca‐Vargas et al. (2016) evaluated the effect of extraction process on the yield of *Bougainvillea* × *buttiana* with seven solvents, and the EY ranged from 1% to 15%. The higher EY obtained in this study may be related to different extraction conditions or the originality of the flower.

**TABLE 1:** Extraction yield (EY), total phenolic (TPC), flavonoid (TFC), and anthocyanin content (TAC) of *Bougainvillea spectabilis* extract (BSE)

Extraction method	EY (%)	TPC (mg GAE/100 g FW)	TFC (mg CE/100 g FW)	TAC (mg CGE/100 g FW)
Shaking	6.12 ± 0.8^c^	2115.76 ± 5.4^c^	946.51 ± 3.7^c^	28.31 ± 2.0^c^
Bath ultrasound	10.56 ± 1.1^b^	2870.83 ± 6.3^b^	1405.39 ± 4.2^b^	69.48 ± 4.2^b^
Probe ultrasound	16.37 ± 1.2^a^	4354.15 ± 5.4^a^	2431.25 ± 3.5^a^	106.57 ± 3.5^a^

*Note:* Different superscript lowercase letters indicate statistically significant difference (*p* < .05).

Abbreviations: CE, quercetin equivalent; CGE, cyanidin‐3‐glucose equivalent; FW, fresh weight; GAE, gallic acid equivalent.

The antioxidant and antimicrobial activity of BSE is related to its high phenolic, flavonoid, and anthocyanin content (Ydjedd et al., 2017). Therefore, evaluating the content of bioactive compounds in the extract is very important. Table 1 shows the content of bioactive compounds in BSE extracted using different methods. Total phenolic content (TPC) of BSE ranged from 2115.76 to 4354.15 mg GAE/100 g FW. The higher and lower total flavonoid content (TFC) was observed in probe ultrasound (2431.25 mg CE/100 g FW) and shaking method (946.51 mg CE/100 g FW), respectively. Compaore et al. (2013) measured the TPC and TFC of *B. spectabilis* and *B. glabra* extract. The higher TPC and TFC were 151.25 mg EGAg^−1^ and 13.30 mg QEg^−1^, respectively (Compaore et al., 2013). In another study conducted by Debiasi et al. (2021), the TPC and TFC of BSE ranged from 32.28 to 52.56 mg EAG.g^−1^ and 4.44 to 5.19 mg Q.g^−1^, respectively. Saleem et al. (2020) measured the TPC and TFC of *B. glabra* obtained using methanol and dichloromethane solvent. The TPC and TFC of methanol extract were 26.04 mg GAE/g and 20.86 mg QE/g, respectively, which was higher than those obtained using dichloromethane solvent. Abarca‐Vargas et al. (2016) reported the TPC ranged from 0.19 to 21.14 mg GA/ g of dry extract obtained from methanolic extract of *Bougainvillea x buttiana*. Kuspradini et al. (2016) reported the content of total phenolics in the extract of four different flowers ranged from 841.85 to 4200 mg GA.g^−1^. They also reported higher TFC and TAC in ethanolic extract of *Jatropha integerrima* flower, which was 1040.66 mg CE/100 g FW and 102.38 mg CGE/100 g FW, respectively (Kuspradini et al., 2016). In this study, a higher TAC (106.57 mg CGE/100 g FW) was observed in the extract obtained from probe ultrasound extraction. Anthocyanins have great potential in the pharmaceutical and food industries because they act as antioxidant agents by donating hydrogen to highly reactive species (Kuspradini et al., 2016).

Anthocyanins are valuable plant‐derived bioactive compounds with high antioxidant activity (Zhao et al., 2017). The results of total anthocyanins content (TAC) of different extracts showed that, similar to TPC and TFC, the higher TAC was observed in the extract obtained by probe ultrasound followed by bath ultrasound and shaking. Sudipta et al. (2012) reported the presence of various secondary metabolites like saponins, flavonoids, terpenoids, and alkaloids in BSE. Many authors reported higher TPC, TFC, and TAC in extracts from the sonication method in comparison to conventional extraction (Razavi & Kenari, 2021). Ultrasound affects the plant matrix by a chain detexturation mechanism in particular order: local erosion, shear forces, sonoporation, fragmentation, capillary effect, and detexturation. In other words, the action of ultrasound cavitation on the matrix resulted in the debris formation. The higher temperature and pressure which is caused by collapsing the microbubbles result in the loosening of the sample cellular membrane and dissolution of bioactive compounds (Aliaño‐González et al., 2020; Huang et al., 2019).

### 3.2 Antioxidant activity

The reactive oxygen species were involved in the expansion of cancer and cardiovascular diseases (Compaore et al., 2013). DPPH and FRAP are two general methods to evaluate the antioxidant activity of most wide materials. The results of antioxidant activity of free and nanoencapsulated extract are shown in Table 2. Free extract showed lower antioxidant activity than encapsulated extract in both evaluating methods. The nanoemulsion prepared at a 1:1 ratio of core to coating exhibited higher antioxidant activity than extract at a 1:2 ratio of core to coating, which is related to the higher amount of BSE. Also, the partial antioxidant activity of the nanoemulsion may be related to nettle seed gum. Zamani and Razavi (2021) evaluated the antioxidant activity between vitamin C and nettle seed gum. Their result revealed that the antioxidant activity of the nettle seed gum at a concentration of 1000 ppm was 38.1% compared to the vitamin C which was 67.9%. They also reported the presence of hydroxyl groups in the nettle seed gum which is responsible for the appearance of radical scavenging (Zamani & Razavi, 2021).

**TABLE 2:** Antioxidant activity of free and nanoencapsulated *Bougainvillea spectabilis* extract (BSE)

Evaluation method	Free extract	1:1	1:2	BHT
DPPH (IC50, mg/L)	486.12 ± 3.31^a^	457.55 ± 4.19^b^	462.93 ± 3.80^b^	32.39 ± 2.26^c^
FRAP (μM Fe^+2^/g)	4.22 ± 0.12^a^	4.09 ± 0.25^b^	4.13 ± 0.32^b^	2.97 ± 0.18^c^

*Note:* Different superscript lowercase letters indicate statistically significant difference (*p* < .05).

The antioxidant activity of BSE was more than those reported by Debiasi et al. (2021) for BSE (DPPH EC_50_ = 2.27 g.g^−1^) and by Gupta & Rathore (2011) for *B. glabra* leave extract (about 90% inhibition), though similar to IC50 reported by Abarca‐Vargas et al. (2016) for *Bougainvillea* × *buttiana* extract which ranged from 223.1 to 1455.68 μg/mL. In a study conducted by Orozco‐Villafuerte et al. (2018), the aqueous extract of *B. spectabilis* exhibited 45.73% and 69.42% of inhibition by the method DPPH and ABTS, respectively. Studies have shown that the antioxidant activity of plant extracts is related to their bioactive content (Kenari et al., 2020; Kuspradini et al., 2016; Orozco‐Villafuerte et al., 2018; Petrova et al., 2016; Razavi & Kenari, 2021; Ydjedd et al., 2017). Orozco‐Villafuerte et al. (2018) observed that there is a direct correlation between the amount of phenolic compounds and antioxidant activity of BSE. In this regard, Petrova et al. (2016) showed a positive linear correlation between total antioxidant activities and total phenolic content in five edible flowers.

Compaore et al. (2013) measured the antioxidant activity of *B. spectabilis* and *B. glabra* extracts using DPPH inhibition, ABTS inhibition, and FRAP methods. They reported the antioxidant ability of extracts in all evaluation methods with a higher significant correlation. Similarly, Saleem et al. (2020) reported the antioxidant activity for *B. glabra* extract in radical scavenging (DPPH and ABTS) and reducing power (FRAP and CUPRAC) assays.

### 3.3 Antimicrobial activity of free and nanoencapsulated BSE

Natural extracts gain more importance in food industries because of nontoxic and ecofriendly properties, as well as renewable and abundant sources from which they are extracted. The MIC and MBC values of a nanoencapsulated BSE in comparison to free extract, reported in Table 3, give a measurement of the activity of the antimicrobial agent against four bacterial pathogens. Both free and encapsulated extracts exhibited antimicrobial activity. Sudipta et al. (2012) reported the antimicrobial activities of different solvent extracts of *B. spectabilis* flowers against different bacteria and fungi. Sidkey (2018) investigated the antibacterial activity of ethanolic extract of *B. spectabilis* leaves against *Bacillus subtilis*, *S. aureus*, *E. coli*, and *Klebsiella pneumoniae*, and the results showed high sensitivity of bacterial strains to extract. Several studies have reported the antimicrobial activity of crude extracts of different plants (Sudipta et al., 2012).

**TABLE 3:** Minimum inhibitory concentration (MIC) and minimum bactericidal concentration (MBC) of free and nanoencapsulated *Bougainvillea spectabilis* extract (BSE) (mg/ml)

Bacteria	Free extract		Nanoencapsulated extract (core to coating ratio)			
			1:1		1:2	
	MIC	MBC	MIC	MBC	MIC	MBC
*Escherichia coli*	750^a^	875^b^	500^a^	625^a^	625^a^	625^a^
*Salmonella enterica*	750^a^	875^b^	500^a^	625^a^	625^a^	625^a^
*Listeria monocytogenes*	625^b^	750^b^	375^b^	500^b^	500^b^	500^b^
*Staphylococcus aureus*	625^b^	750^b^	375^b^	375^b^	500^b^	500^b^

*Note:* Different superscript lowercase letters indicate statistically significant difference (*p* < .05).

The MIC and MBC values of the BSE encapsulated in nanoemulsions always resulted in lower than free extract, therefore suggesting encapsulation of BSE into nanoemulsion has improved the antimicrobial activity by enhancing the transport mechanisms through the cell membrane of target microorganisms. Similarly, the MIC value of encapsulated BSE with a 1:2 core to coating ratio was higher than a 1:1 ratio. Similarly, Donsì et al. (2011) observed that the antimicrobial effects of free and nanoencapsulated d‐limonene and terpenes were more pronounced against gram‐negative bacteria. They also reported higher antimicrobial activity of nanoemulsion compared to none encapsulated ones. Obtained MIC and MBC values are presented in Table 3, showing that the gram‐positive bacteria were more sensitive than the gram‐negative for both free and encapsulated BSE. The main targets of bioactive compounds are the outer membrane of gram‐negative bacteria and the cytoplasmic membrane of microorganisms. Additionally, bioactive compounds could interact with intracellular targets, enzymes, and proteins. Almadiy et al. (2016) exhibited that nanoemulsions prepared from essential oil showed increased antibacterial activity against foodborne bacteria in comparison to pure essential oil. They also revealed that the nanometric‐sized particles might increase the passive cellular absorption mechanisms and reduce mass transfer resistances and increase antimicrobial activities.

### 3.4 Characterization of nanoemulsion

Nanoemulsions are important vehicles for delivering bioactive compounds into various food products, drugs, and nutraceuticals (Almadiy et al., 2016). The particle size of nanoemulsion is a critical parameter closely associated with the aqueous dispersibility, aggregation, and fusion of nanoparticles (Zhao et al., 2017). The results of particle size of different nanoemulsions are shown in Table 4. The particle diameter of samples ranged from 76.5 to 99.3 nm, and significantly increased by increasing the ratio of gum solution. When the amount of gum solution is more than emulsion, a sufficient amount of gum molecules is available to interact with the emulsion droplet, which may result in decreasing the aggregation and reducing the particle size. Rahnemoon et al. (2021) formulated sodium alginate nanocapsules containing pomegranate peel extract at the 2:1 and 4:1 ratio of alginate to extract. They stated a decrease in particle size from 218.9 to 177.8 nm when the alginate ratio increased from 2 to 4. In another study conducted by Kenari et al. (2020), the chitosan:sage seed gum nanoparticles containing Iranian golpar extract were prepared at different extract to wall material ratios (0.10, 0.25, and 0.40) and the particle size decreased when wall material increased. Another reason for bigger particle size and wide size distribution may be due to a higher viscosity of 1:1 nanoemulsion which accelerates droplet aggregation.

**TABLE 4:** Particle size, zeta potential, polydispersity index, and encapsulation efficiency of *Bougainvillea spectabilis* extract (BSE) nanoemulsion

Core to coating ratio	Particle size (nm)	PDI	Zeta potential (mV)	Encapsulation efficiency (%)
1:1	99.3 ± 0.5^a^	0.227 ± 0.01^a^	−31.6 ± 1.5^a^	83.4 ± 0.8^a^
1:2	76.5 ± 0.7^b^	0.198 ± 0.01^b^	−38.2 ± 1.2^b^	76.6 ± 0.4^b^

*Note:* Different superscript lowercase letters indicate statistically significant difference (*p* < .05).

Polydispersity index is a measure of the size heterogeneity of particles in the nanoemulsion. A nanoemulsion is called uniform if the particles have the same size. As Table 4 shows, the PDI of both nanoemulsions was below 0.23. PDI values below 0.2 are usually considered monodisperse, whereas values less than 0.3 are considered ideal. In addition to the smaller particle size of nanoemulsion prepared at the core to coating ratio 1:2, this nanoemulsion exhibited a narrow distribution size. Zhao et al. (20172017) revealed that an increase in anthocyanin concentration caused an increase in particle size and PDI of liposomes, which is in accordance with the result of this study.

Zeta potential indicates the surface charge of nanoparticles and determines the repulsion among charged nanoparticles. The zeta potential results, as a fundamental parameter of the surface charge of the droplets, showed anionic nature for both nanoemulsions (Table 4). The zeta potential of more than +30 mV and lower than −30 mV has good stability. According to the result, the stability of 1:2 nanoemulsions is higher than the 1:1 nanoemulsion. It is attributed to lower zeta potential. Therefore, the properties of nanoemulsions depend on the ingredients that participate in forming the nanoemulsion, encapsulated compounds, dispersing medium, and preparation conditions (Rahnemoon et al., 2021). The negative zeta potential for encapsulated extract in native gums also was reported for Iranian golpar in sage seed gum (Kenari et al., 2020), phenolic compounds of sesame seed in native seed gums (Esmaeilzadeh Kenari & Razavi, 2022), and *Fumaria parviflora* extract in Arabic gum (Razavi & Kenari, 2021).

EE is an important property to describe the effectiveness of the encapsulation process. It points to the possibility of the coating material holding the core substance inside of nanocapsules. The EE ranged from 76.6% to 83.4%. The ratio of core to coating affects EE, and by increasing ratio from 1:2 to 1:1, the EE was increased. It is related to the higher viscosity of emulsion and higher extract concentration. Zhao et al. (2017) reported that higher anthocyanin addition resulted in a significant increase in the EE. Also, Kenari et al. (2020) reported higher EE occurs due to using a larger amount of extract.

### 3.5 Scanning electron microscopy (SEM)

**FIGURE 1:**
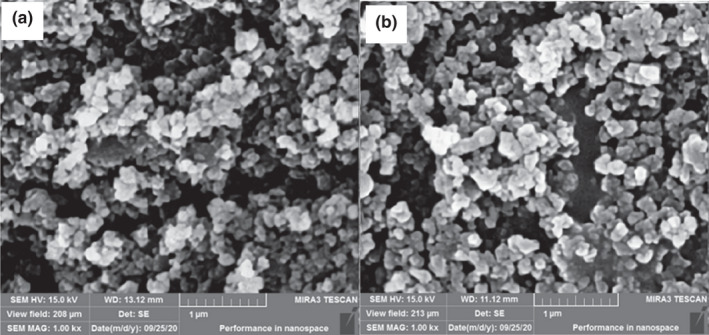
Surface electron morphology of nanoencapsulated *Bougainvillea spectabilis* extract (BSE). Nanoemulsion with core to coating ratio (a) 1:1 and (b) 1:2

Scanning electron microscopy (SEM) images give information about the morphology of nanoparticles and their appropriate surfaces (Ydjedd et al., 2017). Figure 1 presents SEM images of nanoemulsions prepared in different core to coating ratios. The morphology of nanoemulsion with both core to coating ratios was partly spherical in shape. Spherical nanoparticles indicate that coating materials are stable and effectively surround core materials (Razavi & Kenari, 2021). Although the surface of nanoparticles was not uniform, no porose was observed on the surfaces. Minimum agglomeration was observed in nanoparticles, which indicates strong encapsulating properties of nettle seed gum as wall materials. Similar morphology was observed in nanoencapsulated bioactive compounds in gum coatings (Kenari et al., 2020; Razavi & Kenari, 2021; Robert et al., 2010).

### 3.6 In vitro release

**FIGURE 2:**
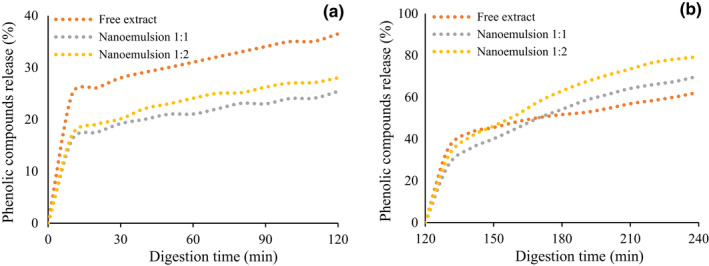
In vitro release of phenolic compounds from free and nanoencapsulated *Bougainvillea spectabilis* extract (BSE). (a) Gastric digestion, (b) small intestine digestion

The digestion process is the first step, before absorption and metabolism, in modulating the bioavailability of bioactive compounds by affecting their bioaccessibility (Ydjedd et al., 2017). Figure 2 shows the release of phenolic compounds during the in vitro gastric and intestinal digestion phase. Generally, the release of phenolic compounds from both nanoemulsion was increased gradually with an extension of the digestion time from 0 to 120 min after digestion in both gastric and intestinal phases. Flores et al. (2015) studied the in vitro release properties of encapsulated blueberry extracts. They found that phenolic contents increased throughout the gastric to the intestinal phase.

The higher total amount of phenolic compounds released by the gastric phase was observed in free extract, followed by nanoemulsion 1:2 and nanoemulsion 1:1, which were 36.5%, 28.03%, and 25.41%, respectively. On the other hand, the final amount of phenolic released by the intestinal phase was 62.36%, 70.13%, and 79.4% from free extract, nanoemulsion 1:1, and nanoemulsion 1:2, respectively. In addition, the release of phenolic from nanoemulsion by the intestinal phase was higher than the release by the gastric phase. This may be because of the strong solubility of coating substances at higher pH of the intestinal phase. This result means that both nanoencapsulated extracts are protected against the condition changes of digestion like enzymes and pH variation. These results revealed that the encapsulated BSE could be effectively absorbed in the small intestine. These results are in accordance with the results of Mohamed et al. (2018), who reported a higher release of betalain from microcapsules in the small intestine phase than gastric phase. However, for free extract, the phenolic compounds are more released in the gastric phase as compared to the nanoencapsulated BSE. These results are in line with the report by Ydjedd et al. (2017), who reported that the release of TPC increased during digestion and TPC in the gastric phase was higher than small intestine phase.

## 4 CONCLUSION

The obtained results demonstrate that BSE in both free and nanoencapsulated forms can scavenge free radicals due to a higher amount of phenolic, flavonoid, and anthocyanin compounds. Additionally, it exhibits antimicrobial activity against *S. aureus*, *E. coli*, *L. monocytogenes*, and *Sa. enterica*. Nettle seed gum could improve the antioxidant and antimicrobial activity of extract. The core to coating ratio 1:1 showed gradual release of bioactive compounds in comparison to ratio 1:2. In conclusion, it could be said that BSE at a concentration of 1000 mg/L, which is nanoencapsulated in *U. dioica* L. seed gum, is a natural functional compound with antioxidant/antimicrobial properties and higher bioavailability. Future research is needed for evaluating the characteristics of fabricated nanoemulsion as natural preservative agents in food products.

## CONFLICT OF INTEREST

Authors declare no conflict of interest.

## ETHICAL APPROVAL

The study does not involve any human or animal testing.

## Data Availability

Research data are not shared.
